# Derivation and validation of the Rapid Assessment of Dementia Risk (RADaR) for older adults

**DOI:** 10.1371/journal.pone.0265379

**Published:** 2022-03-17

**Authors:** Ana W. Capuano, Raj C. Shah, Paul Blanche, Robert S. Wilson, Lisa L. Barnes, David A. Bennett, Zoe Arvanitakis

**Affiliations:** 1 Rush Alzheimer’s Disease Center, Rush University Medical Center, Chicago, Illinois, United States of America; 2 Department of Neurological Sciences, Rush Medical College, Chicago, Illinois, United States of America; 3 Department of Internal Medicine, Rush Medical College, Chicago, Illinois, United States of America; 4 Section of Biostatistics, Øster Farimagsgade, University of Copenhagen, Copenhagen, Denmark; 5 Denmark Department of Cardiology, Herlev and Gentofte University Hospital, University of Copenhagen, Hellerup, Denmark; 6 Department of Psychiatry and Behavioral Sciences, Rush Medical College, Chicago, Illinois, United States of America; University of Kentucky, UNITED STATES

## Abstract

**Background:**

There is no practical dementia risk score in the clinical setting.

**Objective:**

To derive and validate a score obtained by a rapid and simple assessment, which guides primary care providers in predicting the risk of dementia among older adults.

**Design:**

A total of 4178 participants from three longitudinal cohorts (mean age at baseline = 76.8 [SD = 7.6] years), without baseline dementia, followed annually for a median of 10 years (IQR: 5 to16 years, Reverse Kaplan-Meier).

**Participants:**

To derive the score, we used data from 1,780 participants from the Rush Memory and Aging Project (93% White). To validate the score, we used data from 1,299 participants from the Religious Order Study (92% White), and to assess generalizability, 679 participants from the Minority Aging Research Study (100% Black).

**Measurements:**

Clinician-based dementia diagnosis at any time after baseline and predictive variables associated with dementia risk that can be collected in a primary care setting: demographics, clinical indicators, medical history, memory complaints, cognitive and motor tests, and questions to assess functional disability, depressive symptoms, sleep, social isolation, and genetics (APOE e4 and AD polygenic risk score).

**Results:**

At baseline, age, memory complaint, the ability to handle finances, the recall of the month, recall of the room, and recall of three words, were associated with the cumulative incidence of dementia, in the derivation cohort. The discrimination of the RADaR (Rapid Risk Assessment of Dementia) was good for the derivation and external-validation cohorts (AUC_3 years_ = 0.82–0.86), compared to the overall discrimination of age alone (AUC_3 years_ = 0.73), a major risk factor for dementia. Adding genetic data did not increase discrimination.

**Limitations:**

Participants were volunteers, may not represent the general population.

**Conclusions:**

The RADaR, derived from community-dwelling older persons, is a brief and valid tool to predict dementia risk at 3 years in older White and Black persons.

## Introduction

The ability to distinguish persons at high vs. low risk of dementia in the primary care setting could provide a range of benefits and possibly also cost savings, as well as help clinicians better monitor patients and provide focused education about dementia and advance care planning. Dementia, however, is largely underdiagnosed in a large number of persons, and most who meet the criteria do not receive a formal diagnosis in primary care settings [1–4]. This work builds upon previously developed risk scores for dementia, including by borrowing several of the strengths from previous studies and making modifications to overcome some of the limitations. First, we acknowledge that none of the previously developed scores are widely adopted in the primary care setting. This is mostly because they are either not sufficiently brief (taking more than 10 minutes to administer) or not practical (e.g., feasibility of the data gathered) [5, 6]. Among the risk scores studied to date, the few that are brief include a combination of age, education, subjective memory complaint, hypertension, obesity/BMI, stroke, diabetes, cancer, or help needed with finances [7–11]. However, some of these scores also include laboratory or genetic tests (not rapidly obtained) [7, 8]. Others include marital status that, although relevant to many populations, is not relevant to some groups such as for people living in religious communities or less conventional units [10], that we would not like to exclude. Some scores also use medication commonly prescribed such as antidepressants [12], that we prefer not to consider because it practices on prescribing these medications are shown to change over the years [13, 14], including off-label [15], and access to treatment is shown to be biased according to race, ethnicity, and health-care access (e.g. type of coverage) [16, 17]. However, the major novelty of the current risk score is that it combines age with both cognitive and functional measures. Current cognitive risk assessment tools do not include a functional component, which is essential for diagnosing dementia, and do not include a direct assessment of cognitive function. Please note that the proposed score, the Rapid Assessment of Dementia Risk (RADaR), is distinct from the eRADAR, an electronic health record (EHR)-based tool to detect patients with unrecognized dementia [18].

In this study, we developed a practical dementia risk assessment score. We used data from three large longitudinal cohort studies of older persons without dementia at baseline to generate and validate the score. We initially derived the score using data from community-dwelling persons from the Rush Memory and Aging Project (MAP). To externally validate and generalize, we examined the score in mostly White older Catholic clergy (Religious Orders Study, ROS), and in older Blacks (Minority Aging Research Study, MARS). We also evaluated the predictive gain of using genetic information in the practical assessment tool, including APOE e4 status and a polygenic risk score for AD, to help determine whether a gain in prediction would offset the loss from decreased practicality of the tool.

## Methods

This study followed the Transparent Reporting of a Multivariable Prediction Model for Individual Prognosis or Diagnosis (TRIPOD) [19] reporting guidelines and the optimum features of study design and variables selected for dementia risk prediction outlined by Tang et.al. 2015 [20]. Analyses are based on individuals from three ongoing longitudinal clinical cohort studies. MAP began in 1997 and included older-lay persons from the Chicagoland area [21]. ROS began in 1994 and included older Catholic priests, brothers, and nuns, from more than 40 groups across the United States [21]. MARS began in 2004 and included older, lay Black persons in the Chicagoland area recruited from the community and we augmented the cohort with Black participants from the Clinical Core of the Rush Alzheimer’s Disease Core Center [22]. By design, the methods and data collection across the three community-based cohorts are very similar [23], including with very high follow-up rates (>85%), and harmonization of data allow for pooled analyses, as we have published in many prior studies. All studies were approved by an Institutional Review Board of Rush University Medical Center.

At the time of these analyses, 4178 (1872 MAP, 1308 ROS, and 998 MARS) individuals free of dementia and with complete baseline clinical evaluation, completed at least one follow-up evaluation, as needed for the longitudinal analyses proposed here. Across the three cohorts, the average age at baseline was 76.8 years [SD = 7.6] and education was 16.0 years (SD = 3.7), and 75% were women, 28% were Black and 4% were Latino. Participants were followed annually for a median 10 years (Reverse Kaplan-Meier, IQR 5 to16 years). All participants provided written informed consent. The Institutional Review Board of Rush University Medical Center approved each of the three cohort studies.

As we previously published including in these cohorts [24], the polygenic risk score (PRS) was calculated by averaging the risk-increasing alleles weighted by the summary statistic using PRSice-2 software, and scaled by z-scoring. Among the 4178 participants included in the analyses, a total of 3679 (88%) have information on APOE e4 [25] (27% positive), and 3018 (72%) have information on the PRS (mean = 0.0, STD = 1.0, as recently published by our group). Participants with one or two APOE e4 alleles compared to participants without an APOE e4 allele were on average about one year younger (t(4176) = 5.48, Р = 0.03), and had one year less of education (t(4184) = 5.5, Р = <0.0001), and had similar proportions of women (75% vs. 74% χ^2^(1) = 0.136, Р = 0.71) and of Blacks (28% vs. 27%, χ^2^(1) = 0.9, Р = 0.36) and Latinx (4% vs. 4%, χ^2^(1) = 0.098, Р = 0.75). Participants with PRS data compared to participants without were similar in age (t(4184) = 0.07, Р = 0.95), had an average about a half year less education (t(4176) = 3.5, Р<0.001), and had similar proportions of women (74% vs. 76%, χ^2^(1) = 1.44, Р = 0.23) but slightly less Blacks (25% vs. 37%, χ^2^(1) = 62.1, Р<0.0001) and more Latinx (6% vs. 3%, χ^2^(1) = 24.8, Р<0.0001).

The outcome of the study was the occurrence of dementia. Each year, participants had a uniform clinical evaluation that included a structured medical history, detailed neuropsychological testing, and a neurologic examination. Following the evaluation, an experienced clinician classified dementia status according to the criteria of the joint working group of the National Institute of Neurological and Communicative Disorders and Stroke (NINDS) and the Alzheimer’s Disease and Related Disorders Association (ADRDA) [26]. Presence of dementia is determined by a clinician with expertise in making a diagnosis of dementia, after review of performance-based cognitive testing results and neuropsychologist impression, neurological exam data, and interview data. It requires a history of cognitive decline and impairment in at least two cognitive domains, as published previously [21]. Death was considered as a competing risk in analyses.

Candidate predictors were selected based on clinical practicality (e.g., data already routinely collected during a primary care visit, such as a history of hypertension) and plausibility (e.g., based on published literature, such as memory complaints), availability of data in our cohorts, and a detailed review of the literature [20]. Predictors included demographics (age, sex, and education), clinical indicators [27] (measures of blood pressure and body mass index (BMI), clinician diagnosis of stroke, medications, routinely collected blood measures (hemoglobin A1C and total cholesterol level), a medical history (hypertension, smoking, diabetes, head injury, heart problems, and cancer), memory complaints [28, 29], functional disability [30–32] (individual items from three scales: the Instrumental Activities of Daily Living [IADLs], the Katz Index of Independence in Activities of Daily Living [ADLs] and the Rosow-Breslau scale, a composite measure of mobility disability), psychological factors [33, 34] (10 individual items from the Center for Epidemiologic Studies Depression Scale [CESD] and a social isolation score), cognitive testing (individual items from the Mini-Mental State Examination) and motor evaluations [35–37] (chair stand, posture, postural stability, shuffling gait, turning, and body bradykinesia).

### Derivation and external validation

We first calculated the risk score with data from the MAP. A multivariable Fine-Gray sub-distribution hazard regression model was fit to identify factors associated with the cumulative incidence of dementia. The model accounted for death as a competing risk [38]. Time to event was time to dementia, to death, or was censored. The candidate variables were tested in blocks. First, we tested baseline variables routinely collected in a clinical setting including age, sex, education, history of hypertension, diabetes, systolic blood pressure, diastolic blood pressure, pulse pressure, mean arterial pressure, hypertension medications, hemoglobin A1C, cholesterol level, smoking, and history of head injury with loss of consciousness, heart conditions and cancer. Predictors were entered in separate models with a term for age. Significant variables at or below an alpha value or 0.05 were age, BMI, and hypertension. These variables were entered into one model. Using a backward selection and evaluation of direction of effects, age at baseline was the only term remaining. Next, we repeated the process with other variables that are not part of the routine annual visit. We used a stricter alpha of 0.01 since the variables need to be collected additionally during the visit representing additional clinical time. The items that were selected included at baseline subjective memory complaints (using two questions: trouble remembering things; current memory worse than 10 years ago), motor function (body bradykinesia and posture), functional disability (meal preparation, handling medications, handling finances, and shopping), cognitive testing (based on the MMSE: date recall, month recall, city recall, room recall, delayed recall of three words, and writing any complete sentence). Once these variables were identified we performed two additional steps. First, variables were entered into one model per group (i.e. all functional items entered into one model) with a term for age, and were selected using backward elimination. Second, all variables retained were then entered into a single model. After a backward elimination, the final model included: age, memory complaints, handling of finances, month recall, room recall, and three-word delayed recall. As complementary analyses, we also ran the stopped Fine and Gray model [39] at 3, 5, and 10 years with similar results.

**Fig 1:**
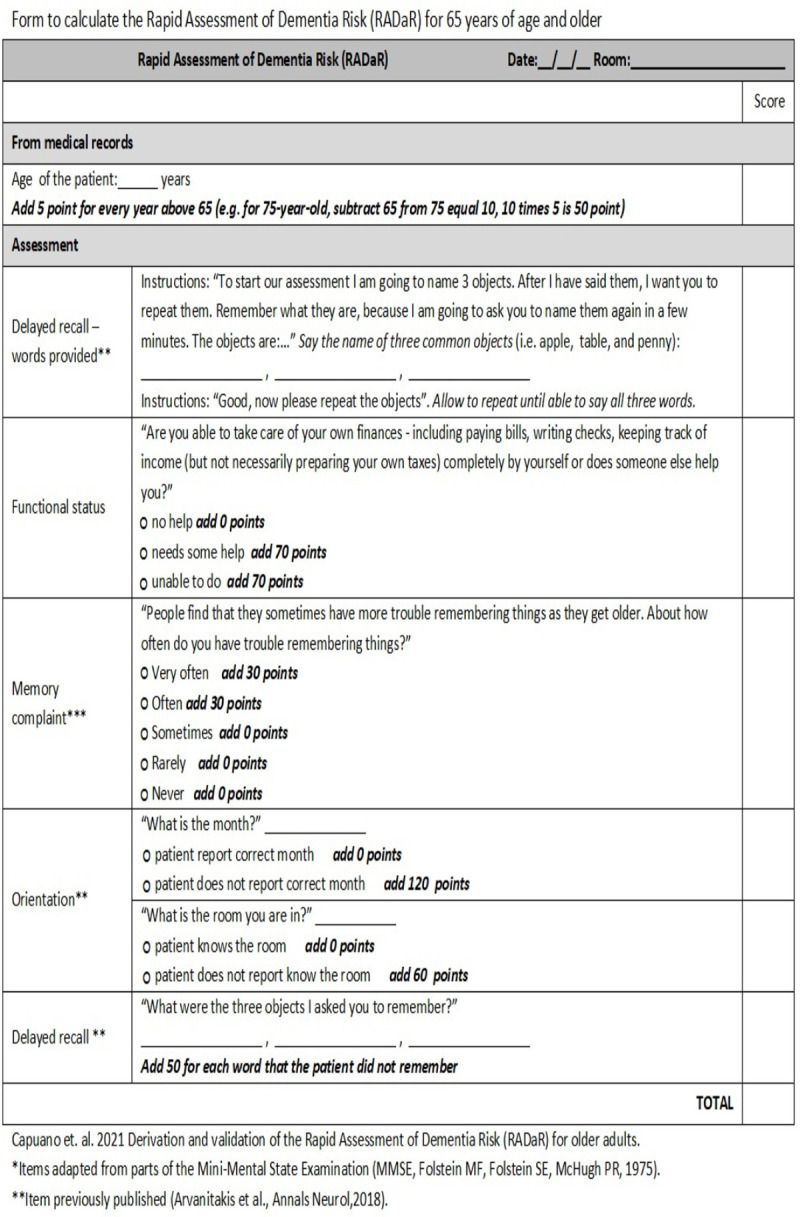
RADaR form. Form to calculate the Rapid Assessment of Dementia Risk (RADaR) for 65 years of age and older.

A points-based risk scoring system for estimating the risk of dementia was created based on the β coefficients in the final model, similar to that suggested by Austin et al. [40]. Fig 1 provides the score sheet for the final components included (without genetic data). This system was applied to the external-validation cohort (ROS) and the generalization cohort (MARS) separately. The final model was rerun with a term for having at least one APOE e4 allele. A points-based risk scoring system for estimating the risk of dementia using genetic data was created based on the β coefficients in the final model. This process was repeated replacing APOE e4 by the PRS [41]. We primarily used the time-dependent-receiver operation curve (ROC) estimated using Inverse Probability of Censoring Weighting (IPCW) to evaluate the performance of the scoring system to predict the time-specific risk [41]. Time-dependent AUC was ascertained using the control defined as a participant that is not a case [42], and 95% confidence interval was computed using bootstrap. The discrimination of the scoring system was compared with the complete scale of the MMSE and age alone. In a secondary analysis, we also compared the discrimination of the scoring system with the Brief Dementia Screening Indicator (BDSI) [12]. The BDSI was calculated for participants below the age of 80 (as recommended), using the following variables, with each being treated as a dichotomous variable other than age: age, education (dichotomized at 12 years), BMI (dichotomized at 18.5 kg/m2), diabetes (y/n), stroke (y/n), IADL (needs help with money or medications: y/n), and depression (medication for depression or reporting that “everything was an effort”: y/n). In additional analyses, we examined the predictive values of the score (including negative and positive predictive values), the discrimination of the scoring system for younger participants (sub-setting to younger than 75 years). We also examined the gain in re-evaluating the patient in the next annual visit by repeating the Fine-Gray competing risk models but with time to event measured from year 1 instead of baseline and terms for the score at baseline. The model included two terms: the RADaR at baseline and the difference between the RADaR at baseline and the first annual follow-up. The improvement in prediction with the additional measure was also evaluated. Analyses were performed in SAS/STAT software, Version 9.4 of the SAS® system for Linux, and in R version 4.0.3 (Survival and Risk Regression packages).

## Results

The baseline characteristics of the participants in the derivation, validation, and generalization cohorts are shown in Table 1. Compared to 1872 participants in the derivation cohort (MAP), the 1308 participants in the validation cohort ROS were different in items related to lifestyle as expected for a religious group, having fewer smokers (19% vs. 42%, χ^2^ (1) = 175.5, *p*<0.0001) for example, and less hypertension (46% vs. 53%, χ^2^ (1) = 16.98, *Р* <0.0001). ROS participants were followed for a median of 18 years based on the Reverse Kaplan-Meier (IQR 6 to 23 years), compared to MAP participants who were followed for a median of 9 years (IQR 5 to14 years), as expected in view of the year of study initiation.

**Table 1:** Characteristics of derivation and validation samples at baseline.

Variables	Derivation	Validation	Generalization
	MAP	ROS	MARS
**Sample size, No.**	1872	1308	998
**Demographics**			
** Age, mean (SD), y**	79.65 (7.46)	75.43 (7.23)	73.22 (6.28)
** Female sex, No. (%)**	1404 (75%)	942 (72.02%)	780 (78.16%)
** Education, mean (SD)**	14.96 (3.27)	18.47 (3.35)	14.92 (3.3)
** Race, No. (%)**			
** White**	1748 (93.38)	1205 (92.13)	0 (0)
** Black**	101 (5.4)	91 (6.96)	998 (100)
** Native American**	8 (0.43)	4 (0.31)	0 (0)
** Pacific Islander**	1 (0.05)	1 (0.08)	0 (0)
** Asian**	7 (0.37)	1 (0.08)	0 (0)
** Other**	2 (0.11)	0 (0)	0 (0)
** Unknown**	5 (0.27)	6 (0.46)	0 (0)
**Medical history at baseline, No. (%)**			
** injury**	109 (6.49%)	99 (7.9%)	48 (5.78%)
** Stroke**	151 (8.72%)	80 (6.45%)	55 (5.52%)
** Hypertension**	992 (52.99%)	596 (45.57%)	765 (76.65%)
** Cancer**	624 (33.33%)	399 (30.5%)	184 (25.27%)
** Diabetes**	235 (12.55%)	175 (13.38%)	253 (25.35%)
** Smoking**	780 (41.71%)	253 (19.34%)	469 (46.99%)
**Clinical at baseline**			
** MCI, No. (%)**	486 (25.96)	331 (25.31)	191 (19.14)
** BMI, mean (SD)**	27.41 (5.35)	27.85 (5.68)	30.52 (6.49)
** Systolic blood pressure, mean (SD)**	134.8 (17.78)	133.46 (18.69)	137.8 (20.73)
** Diastolic blood pressure, mean (SD)**	74.56 (11.25)	74.75 (10.74)	80.95 (11.9)

Compared to the derivation cohort MAP, the generalization cohort MARS was not only different in race composition, per study design, (exclusively Black) but also were on average 6 years younger at baseline (t(2352.1) = 54.46, *Р* = <0.001), had an average of a 3 point higher BMI t(1714.6) = 12.86, *p* = <0.001), had more diabetes (25% vs. 12%, χ^2^(1) = 75.5, *Р* <0.001), more hypertension (77% vs. 53%, χ^2^(1) = 153.51, *Р* <0.001) and more smokers (47% vs. 42% χ^2^(1) = 7.39, *Р* <0.01) but less cancer (25% vs. 33%, χ^2^ (1) = 15.89, *Р* <0.001) and less stroke (6% vs. 9%, χ^2^(1) = 9.32, *Р* <0.01). MARS participants were followed annually for a median of 8 years based on the Reverse Kaplan-Meier (IQR 4 to14 years).

**Fig 2:**
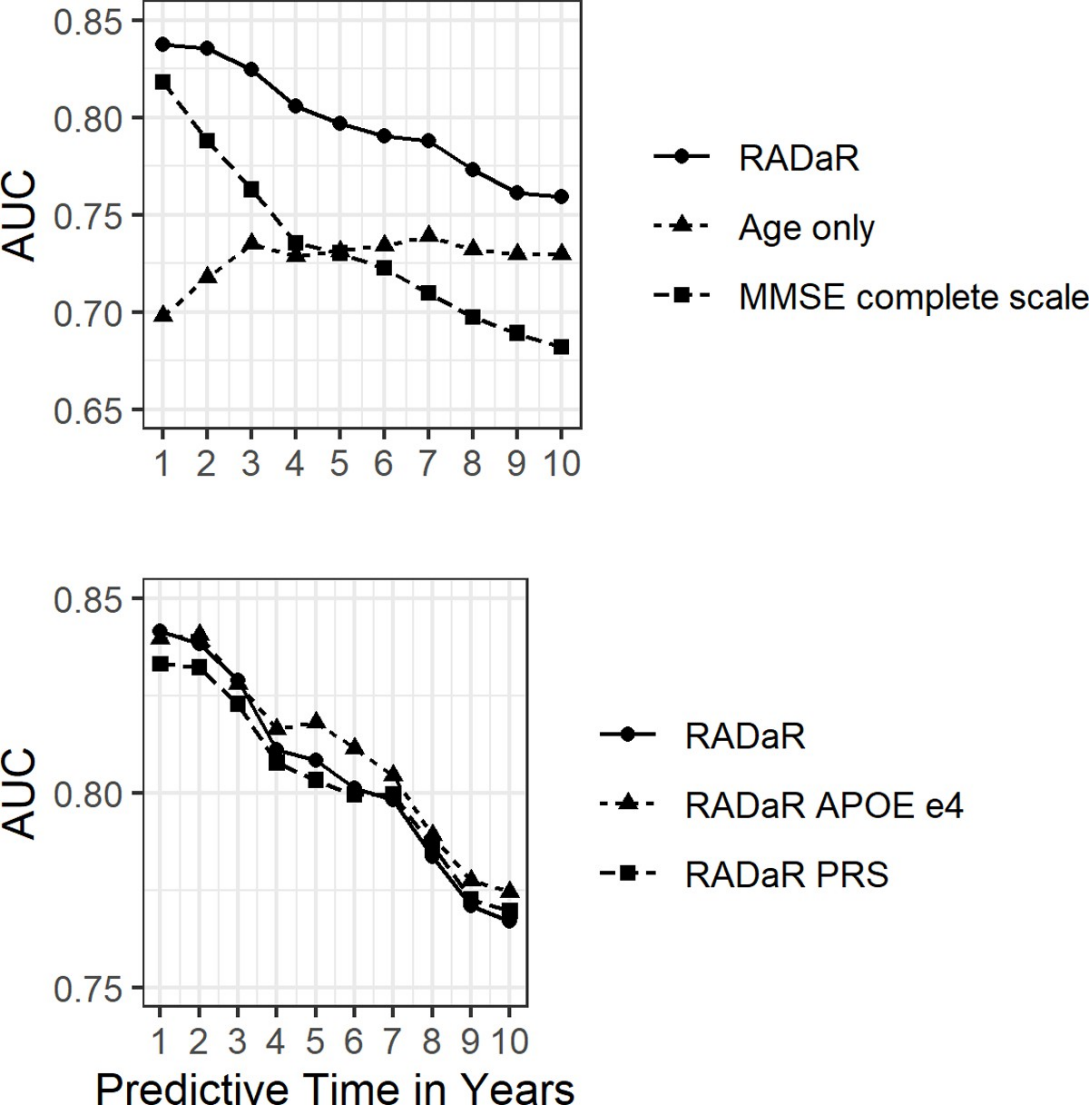
Comparison of AUC for predictive times from 1 to 10 years. RADaR versus age alone and MMSE for the total sample (top) and RADaR with and without genetic information for the sample with genetic information (bottom).

Table 2 shows the results in the derivation cohort from the final Fine-Gray sub-distribution hazard regression model for the cumulative incidence of dementia, and with death (without dementia) as a competing risk. Fig 2 shows the time-specific AUC from 1 to 10 years. The discrimination of the scoring system was also consistently better than using the complete scale of the MMSE (Fig 2). A prediction of 3 years seems adequate for the RADaR, from both a clinical and a statistical perspective. The discrimination of the scoring system at 3 years was good as indicated by the performance for derivation and validation cohorts (AUC_3 year_ = 0.82 to 0.86), as well as all cohorts combined (AUC_3 year_ = 0.83, 95%CI = 0.81, 0.85).

**Table 2:** Fine-Gray sub-distribution hazard regression model for the cumulative incidence of dementia with death as a competing risk with terms for selected predictors and scoring system based on β estimates.

Variable	Derivation cohort				Derivation cohort with APOE e4				Derivation cohort with PRS			
	(n = 1843)				(n = 1584)				(n = 1377)			
	β	SE	*P*	Score System	β	SE	*P*	Score System	β	SE	*P*	Score System
**Age centered at 65**	0.049	0.007	< .0001	5	0.052	0.007	< .0001	5	0.048	0.008	< .0001	5
**Word 1**	0.460	0.192	0.017	50	0.505	0.192	0.009	50	0.425	0.214	0.047	40
**Word 2**	0.506	0.112	< .0001	50	0.475	0.113	< .0001	50	0.434	0.119	0.000	40
**Word 3**	0.472	0.102	< .0001	50	0.460	0.105	< .0001	50	0.502	0.106	< .0001	40
**Month** ^**b**^	1.220	0.249	< .0001	120	1.280	0.223	< .0001	130	1.492	0.275	< .0001	150
**Room** ^**b**^	0.602	0.191	0.002	60	0.493	0.197	0.012	50	0.584	0.208	0.005	60
**Finances** ^**c**^	0.666	0.155	< .0001	70	0.709	0.157	< .0001	70	0.571	0.171	0.001	60
**Memory complaint** ^**d**^	0.280	0.098	0.004	30	0.258	0.099	0.009	30	0.308	0.103	0.003	30
**APOE e4** ^**a**^					0.682	0.102	< .0001	70				
**PRS**									0.110	0.054	0.041	10

β = estimate, SE = standard error, *p* = p-value.

^a^ yes versus no

^b^incorrect vs. correct

^c^help or unable vs. no help

^d^very often and often, versus sometimes, rarely and never.

**Fig 3:**
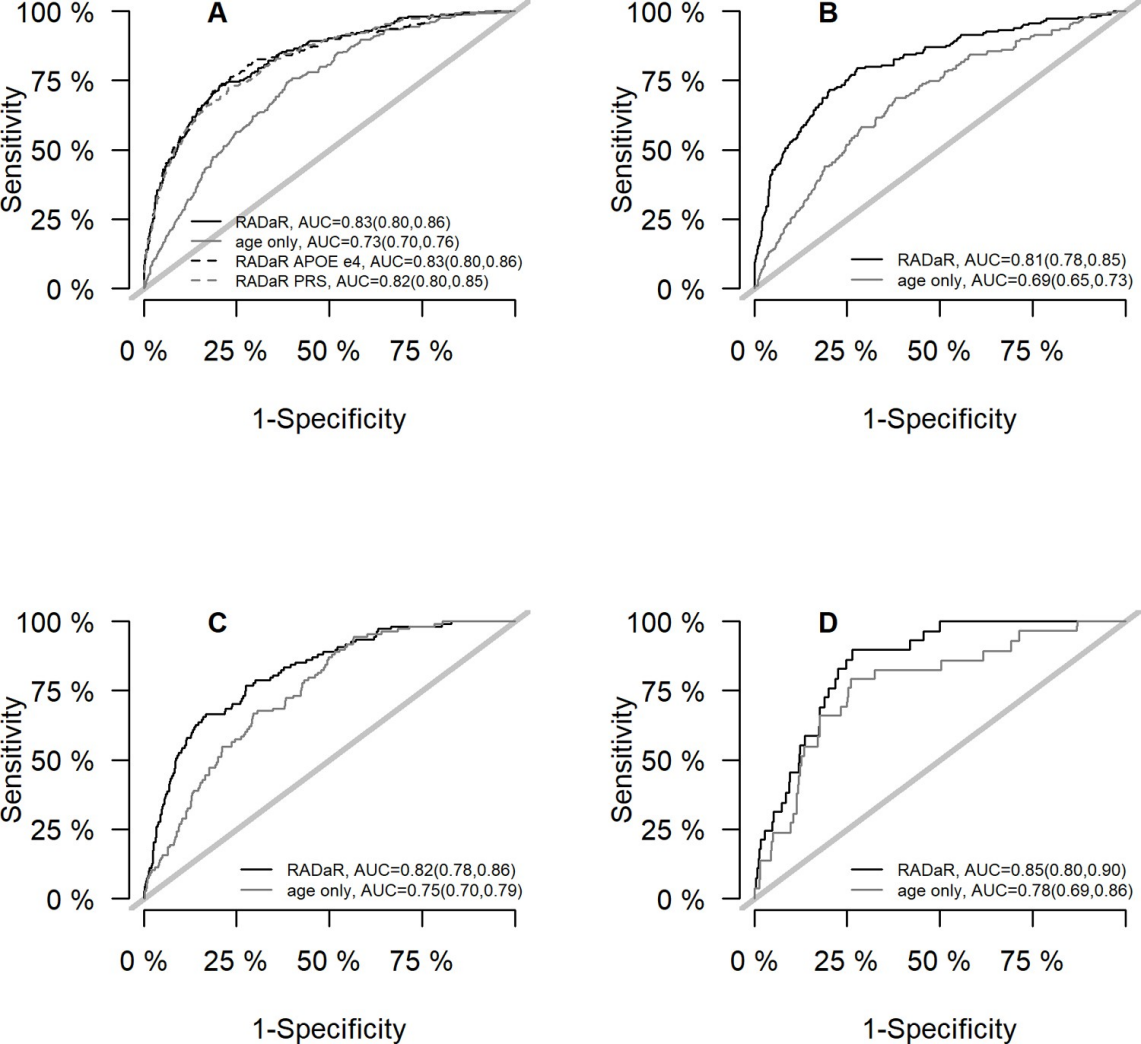
ROC curve and AUC (with 95% CI) of age alone versus RADaR at 3-year prediction time. (A) all with genetics, (B) Derivation cohort, (C) Validation cohort, (D) Generalization cohort.

Because age alone is highly discriminating for dementia, we repeated the analyses with the entire sample to compare the performance of the RADaR with age alone (AUC_3 year_ = 0.74, 95%CI = 0.71, 0.76). Fig 3 compared the ROC curve of age as a sole predicting variable and the RADaR by cohort. Overall, the AUC_3 year_ for age alone was smaller than the AUC_3 year_ for RADaR. Substantially better discrimination performances were found on the derivation, but not in the generalization cohort.

### Genetics

The final Fine-Gray sub-distribution hazard regression model was fitted twice more in the derivation cohort, first adding a term for APOE e4 and second adding a term for PRS (Table 2). The scoring system was redone based on the β estimates. Fig 2A compared the ROC curve of age as a sole predicting variable and the RADaR with and without genetic information. The discrimination of the scoring system updated with genetics had a similar AUC_3 year_ as the scoring without genetics.

### Other comparisons

In secondary analysis we compared the RADaR to the BDSI, a brief tool that also considers age on the continuum. We compared the BDSI to the RADaR and age alone, for the same group of participants (n = 2357) from the three cohorts. The AUC_3 year_ of the BDSI was 0.72 (95%CI 0.67–0.77). By contrast, for this same group the AUC_3 year_ of age alone was 0.68 and of RADaR was 0.82 (respective 95%CI 0.63–0.73 and 0.78–0.86).

### Additional analyses: Predictive values, age of assessment, and frequency of administration

We conducted additional analyses to examine the predictive value of the RADaR. The estimated false positive rate (FPR), true positive rate (TPR), positive predictive value (PPV), and negative predictive value (NPV) of dementia at 3 years considering death as a competing risk for the total sample can be found in the S1 Table. In line with what is found with age alone (e.g. for a baseline age of 80 the NPV = 97% and PPV = 11%), the score provided a high NPV and low PPV. Risk of death is not provided as it is considered as a competing risk, in simple words the model is controlling for it, not estimating it.

To examine age of assessment, first, we studied the discrimination of the RADaR score among younger participants. For that, we used a subset of the data that only included participants younger than 75 years at baseline (n = 1,710), since all three cohorts aim to enroll participants 65 years and older. The discrimination of the scoring system for younger participants was good (AUC _3 years, age<75_ = 0.82, 95% CI = 0.76–0.88).

To assess if there is a gain in re-evaluating the patient in the next annual visit, we examined participants with available data at the first annual follow-up visit (n = 3,826). The Fine-Gray competing risk model had time to event measure from year 1 and two terms: the RADaR at baseline and the difference between the RADaR at baseline and first annual follow-up. The difference in score was a significant predictor (β_baseline RADaR_ = 0.0104, SE = 0.0006, *Р* <0.001, β_yr1-baseline RADaR_ = 0.0053, SE = 0.0007, *Р* <0.001), but the improvement in prediction was very small, slightly increasing the AUC_3 year_ from 0.83 to 0.84 (95%CI = 0.81, 0.86).

## Discussion

In this study, we developed and validated a practical, including brief, simple, and comprehensive, risk score for predicting dementia in older White and Black adults. The RADaR includes only five questions and current age. The five questions are only asked of the individual being evaluated, and pertain to the presence of memory complaints, difficulty with handling finances, orientation to month and room (one question each), and delayed recall of three words|. These five questions should take less than five minutes to have answered. The discrimination of the scoring system at 3 years was good (AUC_3 year_ = 0.82 to 0.86) in comparison to equivalent and more complex risk score and predictive models (ranging from 0.7 to 0.932, Hou et al. 2018). Altogether, the RADaR should take less than 10 minutes to administer, calculate the total score, and determine the level of dementia risk.

We evaluated the need for testing in the younger old (<75 years old), retesting after one year, and the utility of adding genetic information such as the presence of an APOE e4 allele. The score has a good discrimination among persons older than 65 years, including for those between 65 and 75 years old (“younger old”). The retest after one year had a very small improvement in prediction. This finding should be further explored because it can represent significant savings for healthcare, and specifically Medicare, by redirecting limited healthcare resources toward other preventive programs for older adults, including for dementia and other purposes. Finally, we did not find a consistent gain in prediction by the addition of genetic information, including the well-established APOE e4 gene for sporadic Alzheimer’s Disease, as well as the newly developed PRS for dementia. Given that genetic tests should be done with adequate medical counseling, are not always readily available, add to the time and cost of the assessment, and some patients are resistant to the idea of genetic testing, a risk score which is not dependent on genetic information is more practical in primary care settings.

In this work, our goal was to provide the primary care physician and other clinicians with a quick (<10 minutes), objective, practical yet comprehensive risk assessment for dementia. We focused on a tool for prediction, rather than on screening of dementia. Several studies published in the literature have proposed tools for screening. For example, among the most widely used is the Mini-Mental State Examination (MMSE) [37]. The MMSE has 30 questions, takes about 10 minutes, is not free, is better applied with standardized methods including by trained testers, and when used alone in our data (for example not considering age and other important variables), has lower discrimination which would not be adequately informative for the clinician. Further, some studies suggest certain questions may not operate the same for diverse populations or those with low education (e.g., Leveille et al. 1998) [43]. Our clinical prediction tool differs from clinical screening tools by including clinical and demographic factors known to be associated with dementia risk, among which primarily is age. Indeed, age as a sole variable is highly discriminating. The tool closest to ours of which we are aware and could be used for prediction, is the Brief Dementia Screening Indicator (BDSI) [12], which is brief and considers age on the continuum. The BDSI had some gain in comparison to age only, but the RADaR showed consistent greater discrimination than both the BDSI and age separately. Note that BDSI also considers education, a demographic variable that was not important in the RADaR and did not make the first cut in the selection process of our work and has lower discrimination.

With about 49.2 million adults aged 65 years and older in the US CENSUS of 2018 and the proportion of elderly in the population rising in the decades to come, [44] having a brief predictive tool for dementia presents several advantages. Indeed, during the annual wellness visit for which a cognitive assessment is already recommended in the elderly [4], our tool would provide added information and in a rapid and reliable way. Identification of persons at low risk for dementia in the primary care setting is very important because of the increasing number of screenings the physician is required to administer and monitor in the clinic. Although this tool was not intended as a screening, the negative predictive value of the RADaR is estimated at 100% for a score below 60, so the tool could serve to identify persons where dementia screening could be spaced further apart. For instance, the high negative predictive value facilitates decision-making by the clinician regarding persons with a low likelihood of dementia in 3 years and for whom utilizing an appropriate screening tool for dementia [45] can potentially be postponed for two years. Thus, in addition to reassuring the patient about their dementia risk, the amount of time saved during primary care visits could be put to other uses than cognitive screening. In contrast, a patient who is not at low risk, or who presents new symptoms during the annual well visit, should be followed more closely and possibly directed for more formal dementia screening, evaluation and monitoring.

Identification of persons at low risk for dementia is important in the patient perspective. It is a positive message to give patients who are concerned about dementia. Patients’ concerns are well justified. While it is estimated that 5.8 million people live with Alzheimer’s Disease dementia in the US alone [46], there is still no cure and preventive measures are largely not yet shown to be efficacious in large scale phase 3 clinical trials [47]. Further, the disease has a high burden on individuals, families, and society, and the earlier it presents itself the worse the burden, not only emotionally but on practical matters such as family finances. That is especially true because an increasing number of seniors are still in the workforce, according to CENSUS [44]. Particularly in the group of 65 to 74-year-old persons, about 30% of males and 22% of females are still working [44].

The RADaR can be applied to persons at least 65 years old to assist with predicting the risk of dementia. We are also making a calculator available at the Rush Alzheimer’s Disease Center Research Resource Sharing Hub at https://www.radc.rush.edu. Tabulating the score is simple. For example, an 70-year-old patient who needs help writing checks and often has trouble remembering things but recalls the month, the room he/she is in, and three separate words, will have a RADaR of 125 points: 25 points for age, 70 points for functional problems, and 30 points for memory complaints.

While other risk factors for dementia have been found, including female sex, other vascular risk factors such as diabetes and brain conditions such as traumatic brain injury, and psychosocial and environmental factors, among many more, these did not contribute to the RADaR. Therefore, the primary care physician will need to continue to use good clinical judgement in the evaluation of an individual patient and in decision-making, including whether to do a formal dementia screen and whether to suggest any preventative care recommendations.

### Strengths and limitations

This study has important strengths. We used large, well-characterized community-based cohorts. Follow-up was annual, over several years, and there was a high rate of participation, reducing the chance of bias due to selective attrition. We also used standardized diagnostic classification for dementia. The derivation and validation cohorts were very distinct, creating a challenging validation system and further supporting the results. The validation/generalization groups included large numbers of Blacks and participants living a very distinct lifestyle, specifically religious living communal lives. We also used established predictors that were collected annually and allowed us to study the importance of change in scores. A recent review on predictive models that included risk scores [5], stated that limitations of these models include the fact that most risk scores have been developed in Whites only, few have been externally validated and most ignore the feasibility of getting the information to calculate the risk score. Another strength of this work is the contrast of the performance of the score with age alone, rarely done by other works developing risk scores.

An important methodological limitation is that participants were highly educated research volunteers not representative of the general population, so results may vary in other groups, including in other minority populations (i.e. Latinx) and populations with lower educational attainment. Also, this tool was developed in a high-income country (the United States) and may be less applicable in other health care delivery systems in the world. For low- and middle-income countries, validated tools do exist and have been tested [11]. Further studies may be needed for generalization of the RADaR in cohorts from other countries to see how well it performs. In addition, while the RADaR score was generated, validated and generalized in cohorts with very different characteristics and was designed to be practical for primary care, it still needs to be validated in in the clinical setting. That is an important and necessary next step which we hope will be implemented. Finally, our findings need to be further contextualized and studied in the broader space of the initial dementia work-up, recognizing that dementia pre-screening, screening, diagnosis, management and monitoring is a complex and lengthy process, for which many tools are necessary. Also, there are limitations in the use of prediction tools for dementia. The U.S. Preventive Services Task Force did not find strong evidence for harm (i.e. labeling of the individual and the unintended effects of a false-positive result) caused by dementia screening [47, 48]. Such undesirable effects can be observed for predictive scores as well.

## Supporting information

S1 Table The false positive rate (FPR), true positive rate (TPR), positive predictive value (PPV), and negative predictive value (NPV) of dementia at 3 years considering death as a competing risk for the total sample (all cohorts). Predictive value of the RADaR at 3 years considering death as a competing risk based on all cohorts combined are provided.
(DOCX)
