# Supplementary material for: Derivation and validation of the Rapid Assessment of Dementia Risk (RADaR) for older adults
Source: PLoS One. 2022 Mar 17;17(3):e0265379. doi: 10.1371/journal.pone.0265379 (PMC8929636; doi:10.1371/journal.pone.0265379)
Supplement: S1 Table — Predictive value of the RADaR at 3 years considering death as a competing risk based on all cohorts combined are provided. (DOCX) [file pone.0265379.s001.docx]

| S1 Table. The False Positive Rate (FPR), True Positive Rate (TPR), Positive Predictive Value (PPV), and Negative Predictive Value (NPV) of dementia at 3 years considering death as a competing risk for the total sample (all cohorts) | | | | |
| --- | --- | --- | --- | --- |
| RADaR* | FPR | TPR | PPV | NPV |
| 0 | 97.7% | 100% | 6% | 100% |
| 10 | 93.1% | 100% | 6.3% | 100% |
| 20 | 88.8% | 100% | 6.6% | 100% |
| 30 | 83.7% | 99.5% | 6.9% | 99.8% |
| 40 | 77.8% | 98.2% | 7.3% | 99.5% |
| 50 | 71.6% | 97.3% | 7.8% | 99.4% |
| 60 | 65.1% | 96.8% | 8.5% | 99.4% |
| 70 | 58.3% | 95.9% | 9.3% | 99.4% |
| 80 | 51.1% | 91.8% | 10.1% | 99% |
| 90 | 44.4% | 90% | 11.3% | 98.9% |
| 100 | 38.3% | 86.8% | 12.4% | 98.7% |
| 110 | 32.7% | 82.8% | 13.7% | 98.4% |
| 120 | 28% | 81.9% | 15.5% | 98.4% |
| 130 | 23.3% | 78.2% | 17.4% | 98.3% |
| 140 | 19.9% | 73.2% | 18.7% | 98% |
| 150 | 16.4% | 70% | 21.1% | 97.8% |
| 160 | 13.8% | 64.6% | 22.6% | 97.5% |
| 170 | 11.3% | 61% | 25.3% | 97.3% |
| 180 | 9.6% | 55% | 26.5% | 97% |
| 190 | 7.8% | 51% | 29.1% | 96.8% |
| 200 | 6.4% | 45.5% | 30.8% | 96.5% |
| 210 | 5% | 42.9% | 35.1% | 96.4% |
| 220 | 4.1% | 36.5% | 35.9% | 96% |
| 230 | 3.3% | 32.5% | 38.2% | 95.8% |
| 240 | 2.5% | 29.7% | 42.8% | 95.7% |
| 250 | 2.1% | 25.7% | 43.8% | 95.5% |
| * Mean (SD)= 113 (72), median (IQR)=102 (60-152) | | | | |
